# The impact of community-acquired critical sepsis on long-term mortality and morbidity—a nationwide cohort study

**DOI:** 10.1038/s41598-026-53619-9

**Published:** 2026-05-20

**Authors:** Ann-Charlotte Lindström, Jesper Eriksson, Mikael Eriksson, Erik von Oelreich, Johan Mårtensson, Emma Larsson, Anders Oldner

**Affiliations:** 1 Perioperative Medicine and Intensive Care, Karolinska University Hospital Solna, 171 76 Stockholm, Sweden; 2 Section of Anaesthesiology and Intensive Care Medicine, Department of Physiology and Pharmacology, Karolinska Institutet, Stockholm, Sweden; 3 Department of Anaesthesia, Operation and Intensive Care, Uppsala University Hospital, Uppsala, Sweden; 4 Department of Surgical Sciences, Anaesthesia and Intensive Care, Uppsala University, Uppsala, Sweden

**Keywords:** Diseases, Health care, Medical research, Risk factors

## Abstract

The long-term consequences of sepsis, including mortality and morbidity among survivors, are insufficiently understood. This nationwide matched cohort study investigated the association between presumed community-acquired critical sepsis (PCACS)and long-term mortality, the morbidity of burden before and after ICU admission, and causes of death. Patients admitted to Swedish ICUs for PCACS over a ten-year period (*n* = 10,072) were matched 1:5 with population controls (*n* = 50,180). Data on demographics, comorbidities, education, income, and causes of death were collected. Conditional Cox regression was used to assess long-term mortality. Patients with sepsis exhibited a progressive increase in morbidity during the five years preceding ICU admission, which further escalated in the year following discharge. Sepsis was associated with excess mortality up to three years post-admission. Among one-year survivors, sepsis remained independently associated with increased mortality between years one and three, even after adjusting for prior morbidity, indicating persistent long-term effects not fully explained by diagnosed conditions. Early deaths were primarily due to infections and cancer, while later deaths were dominated by cancer and circulatory diseases. These findings highlight the sustained impact of sepsis on survivors, underscoring the need for long-term follow-up and targeted interventions to mitigate post-septic morbidity and mortality.

**Supplementary Information:**

The online version contains supplementary material available at 10.1038/s41598-026-53619-9.

## Introduction

Sepsis poses a significant global health challenge^1^, with an estimated 50 million cases annually and accounting for almost 20% of all global deaths^2^. Furthermore, the incidence of sepsis is reported to be on the rise^3–7^. As such, sepsis is a common cause of ICU admission and is reported to be the most frequent cause of death in this setting^8^. With advances in intensive care medicine, the number of ICU survivors has been reported to increase^9,10^. The trajectory of these survivors has become a focus as emerging data indicate poor long-term outcomes in terms of morbidity, quality of life, and survival^11–13^. The contributing factors to these outcomes remain to be elucidated.

To what extent exposure to sepsis is associated with excessive long-term mortality is not fully explored, nor are the actual causes of death and the patterns of morbidity in the aftermath of sepsis. Most patients with sepsis admitted to an ICU carry a significant burden of comorbidities, potentially influencing their outcomes. Moreover, the trajectory and long-term consequences for patients without significant medical conditions on admission are poorly investigated, offering limited insight into the direct long-term effects of sepsis per se.

The aim of this nationwide matched cohort study was to investigate the association between presumed community-acquired critical sepsis and long-term mortality. Moreover, we sought to examine the burden of morbidity before and after ICU admission and to analyse the causes of death. In addition, these parameters were also be explored among the subgroup of patients with sepsis without significant medical conditions at ICU admission.

## Materials and methods

### Ethics approval

This nationwide matched cohort study was approved by the regional ethical review board in Stockholm, Sweden (approval numbers 2018/725 − 31/2 and amendment 2024-01991-02) and waived the requirement for informed consent. The study adhered to the Strengthening the Reporting of Observational Studies in Epidemiology (STROBE) recommendations for cohort studies^14^. All research was conducted in accordance with national guidelines and regulations.

### Study population

Community-acquired critical sepsis was defined as a patient diagnosed with sepsis and admitted to the ICU directly from an emergency department (ED) without documented recent healthcare exposure identifiable in our data. This is a pragmatic proxy for community acquisition rather than a definitive confirmation. Patients admitted via the ED may still have recent healthcare exposure, including recent hospitalisation, transfer from another facility, or residence in long-term care therefore the term presumed community-acquired critical sepsis (PCACS) was used. All admissions to ICUs in Sweden from January 1, 2008, to December 31, 2017, with either a primary diagnosis of sepsis (ICD-10 codes A41.9, R57.2, and R65.1) or a primary diagnosis of an infectious disease (e.g., bacterial meningitis) with sepsis as a secondary diagnosis and admitted to the ICU directly from the emergency department were identified through the Swedish Intensive Care Register (SIR). For patients with several admissions, only the first admission was included in the study.

For each sepsis case, the government agency Statistics Sweden selected five individuals from the population, not admitted to an ICU for sepsis at the time of the index date, as controls from the Swedish Register of Total Population. Controls were matched on age, sex, and county of residency on the calendar day of ICU admission for the corresponding sepsis patient^15^. In cases, follow-up time was calculated from the day of ICU admission day to death or at the end of the follow-up time of three years. For controls, follow-up time was calculated from the calendar day of the corresponding case´s ICU admission day to death or at the end of the follow-up time of three years. The admission day for cases and corresponding calendar day for control individuals are referred to as the index date.

### National register data

The Swedish Intensive Care Register is a national quality register that collects individual patient data from all Swedish ICUs and operates within the legal framework of the Swedish National Quality Registers. Written informed consent is not required, but the patient may withdraw their data from the register at any time. The register contains data on baseline demographics, variables included in the Simplified Acute Physiology Score (SAPS) III and the Acute Physiology and Chronic Health Evaluation (APACHE) II and mortality. Data are recorded in raw format and transferred electronically to SIR after local validation. After central validation at SIR, incomplete or inconsistent (entries outside pre-specified limits) patient records are returned to the specific ICUs for correction before data are added to the master database. In 2021, the registry achieved complete coverage (100%) of intensive care departments in Sweden, compared to 95% in 2017 and 84% in 2008^16^. Estimated Mortality Rate (EMR) was based on APACHE II for patients included until 2012 and thereafter based on SAPS III.

The National Board of Health and Welfare (NBHW) administers several registries in Sweden^17^. Data about co-morbidities were acquired from the Swedish National Patient Register up to five years prior to ICU admission and two years after. The registry holds information on in-patient and out-patient care episodes, including ICD-10 codes. Primary care is not included in the register. Somatic co-morbidities were classified by Charlson’s Comorbidity Index (CCI)^18^.

Information about the date and causes of death was obtained from the Cause of Death Register and classified according to ICD-10 codes. The Cause of Death Register has a missing data rate of less than 1.5% for underlying causes of death and less than 2% for date of death^19^. Data on education and income were extracted from the Longitudinal Integration Database for Health Insurance and Labour Market Studies (LISA), managed by Statistics Sweden^20^. Level of education was categorised as ≤ 9 years, 10–12 years, and > 12 years of schooling, respectively, the last category equalling university level. Income was categorised into three groups: low, moderate, and high. Low income was defined as an income < 50%, and high income as an income ≥ 200% of the median income in Sweden the calendar year before ICU admission.

Psychiatric illness and substance abuse were defined as the presence of a diagnosis in ICD groups F20-F99 and F10-F14, F16 and F18-F19, respectively. The Swedish Prescribed Drug Register, managed by the NBHW, contains information on all prescribed dispensed drugs in Sweden. It includes personal identity numbers from 1 July 2005, thus enabling linkage to other registries from that date. The register is considered to have 100 per cent coverage; all dispensed drugs that require a prescription are included^21^.

Immunosuppressive therapy was defined as having filled at least one prescription with Anatomical Therapeutic Chemical Classification (ATC) codes A07EA06, H02A, H02B, L01, L03A, L03B, L04A in the four months preceding the index date. For steroids (codes A07EA06, H02A, H02B) we further required that the total defined daily doses (DDD) during the four months exceeded 60 DDD.

### Outcomes

The primary outcome was all-cause mortality during the follow-up period of three years from the index date. Secondary outcomes were causes of death and morbidity before and after exposure to PCACS.

### Statistical analysis

Characteristics of the study cohort are presented as proportions and percentages for categorical data. Continuous data are presented as median with interquartile ranges (IQR). Survival curves of cases and controls were estimated using the standard Kaplan–Meier estimator and compared between groups by a log-rank test. Due to low levels of missingness (< 2%, please see results), complete case analyses were performed. All individuals were censored at three years after the index date. Hazard ratios (HRs) for mortality, comparing patients admitted with sepsis to controls, were estimated using multivariable conditional Cox regression analysis. HRs were presented with 95% confidence intervals. The proportional hazards assumption was tested using Schoenfeld residuals and log-log plots.

Conditional Cox proportional regression models were used to examine the associations between exposure to PCACS and all-cause mortality during the first three years after the index date, adjusted for potential confounders selected a priori (CCI, psychiatric disease, substance abuse, immunosuppressive therapy as well as income and level of education). Since there were indications of a violation of the proportional hazards assumptions for the first year after sepsis, we accommodated the time-varying effect of sepsis on mortality by extending the Cox model, allowing the effect of sepsis to vary over time. The time-varying coefficient model was fit by specifying an interaction between sepsis and a function of time. We subsequently calculated HR for 3, 6, 9, 12, 18, 24, 30, and 36 months after the index date.

In addition, we examined the association between sepsis and mortality one to three years after the index date in one-year survivors. In this additional exploratory analysis, we adjusted for CCI (also considering “new” morbidities recorded within one year after the index date as well as immunosuppressive drugs prescribed within four months of the one year date) and for other confounders mentioned above.

A two-sided P-value of < 0.05 was used to indicate a statistically significant difference. All statistical analyses were performed with Stata/MP 16.1 (StataCorp, College Station, TX).

## Results

### Demography

The study population included 10 072 patients with sepsis and 50 180 controls. Characteristics of the study population are presented in Table 1. The matching variables of age and sex were well-balanced. The median age was 70 years (IQR 60–78) and the majority was male (58%). Approximately half (52%) of the patients with sepsis had a CCI of ≥ 2, compared with only 18% in the control group. The sepsis cohort had an EMR of 0.28 (IQR 0.14–0.47). High income and education levels were more common among control individuals.

**Table 1:** Baseline characteristics and mortality of patients with sepsis and matched controls.

	Controls	Patients with sepsis
	*n*=50,180	*n*=10,072
Age	70 (60–78)	70 (60–78)
Sex (male)	58% (29,175)	58% (5863)
Charlson’s Comorbidity Index		
0	69% (34,748)	31% (3154)
1	12% (6256)	16% (1647)
≥2	18% (9176)	52% (5271)
Psychiatric disease	5% (2628)	15% (1525)
Substance abuse	2% (812)	9% (930)
Education		
Low	33% (16,729)	41% (4107)
Medium	41% (20,472)	41% (4105)
High	24% (12,237)	16% (1637)
Missing	1% (742)	2% (223)
Income		
Low	11% (5719)	14% (1412)
Medium	82% (41,061)	82% (8250)
High	7% (3330)	4% (382)
Missing	0% (70)	0% (28)
90-day mortality	1% (396)	32% (3272)
1-year mortality	3% (1653)	41% (4157)
3-year mortality	9% (4503)	52% (5250)
Estimated mortality ratio	n/a	0.28 (0.14–0.47.14.47)

General admission characteristics and mortality of patients with sepsis and their reciprocal controls. Estimated Mortality Rate (EMR) was calculated using acute Acute Physiology And Chronic Health Evaluation II (APACHE II) for patients included before 2013 or Simplified Acute Physiology Score III (SAPS III) from 2013 onwards, representing the predicted probability of in-hospital death based on illness severity at ICU admission. Data presented as median (IQR) for continuous measures, and % (n) for categorical measures. n/a, not applicable.

Almost one third of the patients with sepsis had a CCI of zero on admission. This group had a median age of 65 (IQR 50–75), a male predominance, and an EMR of 0.21. Levels of education and income showed similar distributions as in the entire sepsis cohort (see Table 2). Small numbers (< 2%) of missing data were noted for education and income variables.

**Table 2:** Baseline characteristics and mortality of patients with sepsis and matched controls with Charlson’s Comorbidity Index zero at ICU admission.

	Controls	Patients with sepsis
	*N* = 11,901	*N* = 3154
Age	61 (46–71)	65 (50–75)
Sex (male)	54% (6422)	55% (1748)
Charlson’s Comorbidity Index		
0	100% (11,901)	100% (3154)
Psychiatric disease	5% (574)	14% (426)
Substance abuse	1% (155)	8% (242)
Education		
Low	25% (2987)	37% (1180)
Medium	44% (5269)	41% (1296)
High	29% (3461)	18% (582)
Missing	2% (184)	3% (96)
Income		
Low	13% (1552)	16% (501)
Medium	79% (9411)	79% (2501)
High	8% (904)	4% (137)
Missing	0% (34)	0% (15)
90-day mortality	0% (14)	24% (755)
1-year mortality	1% (97)	28% (872)
3-year mortality	3% (337)	34% (1071)
Estimated mortality ratio		0.21 (0.09–0.40)

General admission characteristics and mortality of patients with sepsis with Charlson’s Comorbidity Index zero at ICU admission and reciprocal controls with Charlson’s Comorbidity Index zero at the index date. Estimated Mortality Rate (EMR) was calculated using acute Acute Physiology And Chronic Health Evaluation II (APACHE II) for patients included before 2013 or Simplified Acute Physiology Score III (SAPS III) from 2013 onwards, representing the predicted probability of in-hospital death based on illness severity at ICU admission. Data presented as median (IQR) for continuous measures, and % (n) for categorical measures. n/a, not applicable.

### Comorbidity trajectories

**Fig. 1:**
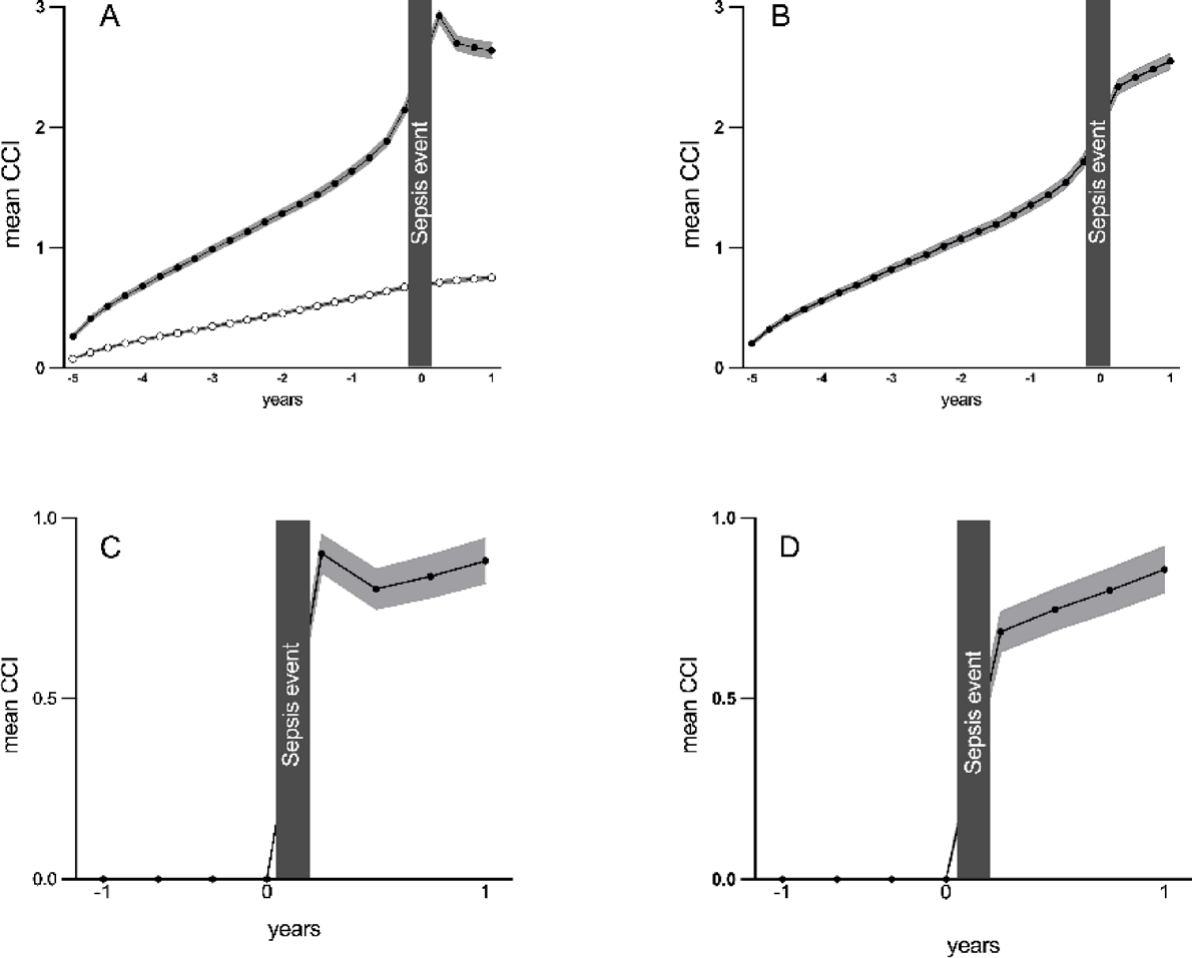
Mean Charlson’s Comorbidity Index (CCI) over time. (**A**) Patients with sepsis (black circles) and control individuals (open circles). (**B**) Patients with sepsis surviving ≥ 1 year after admission. (**C**) Patients with sepsis with CCI zero before admission. (**D**) Patients with sepsis with CCI zero before admission surviving ≥ 1 year after admission. 95% confidence intervals shown as shades.

 Mean CCI increased over the five-year observation period preceding the index date (see Fig. 1A). This increase was more pronounced among patients with sepsis compared to controls. Moreover, the increase was further accentuated during the year preceding ICU admission for patients with sepsis. On admission, the most prevalent comorbidities among patients with sepsis were diabetes, cancer, and congestive heart failure (see supplemental Table). Charlson’s comorbidity index continued to increase during the following 12 months among ICU survivors (see Fig. 1B). The apparent decrease in mean CCI after the index date in Fig. 1A C reflects selective mortality among patients with higher comorbidity burden.

**Fig. 2:**
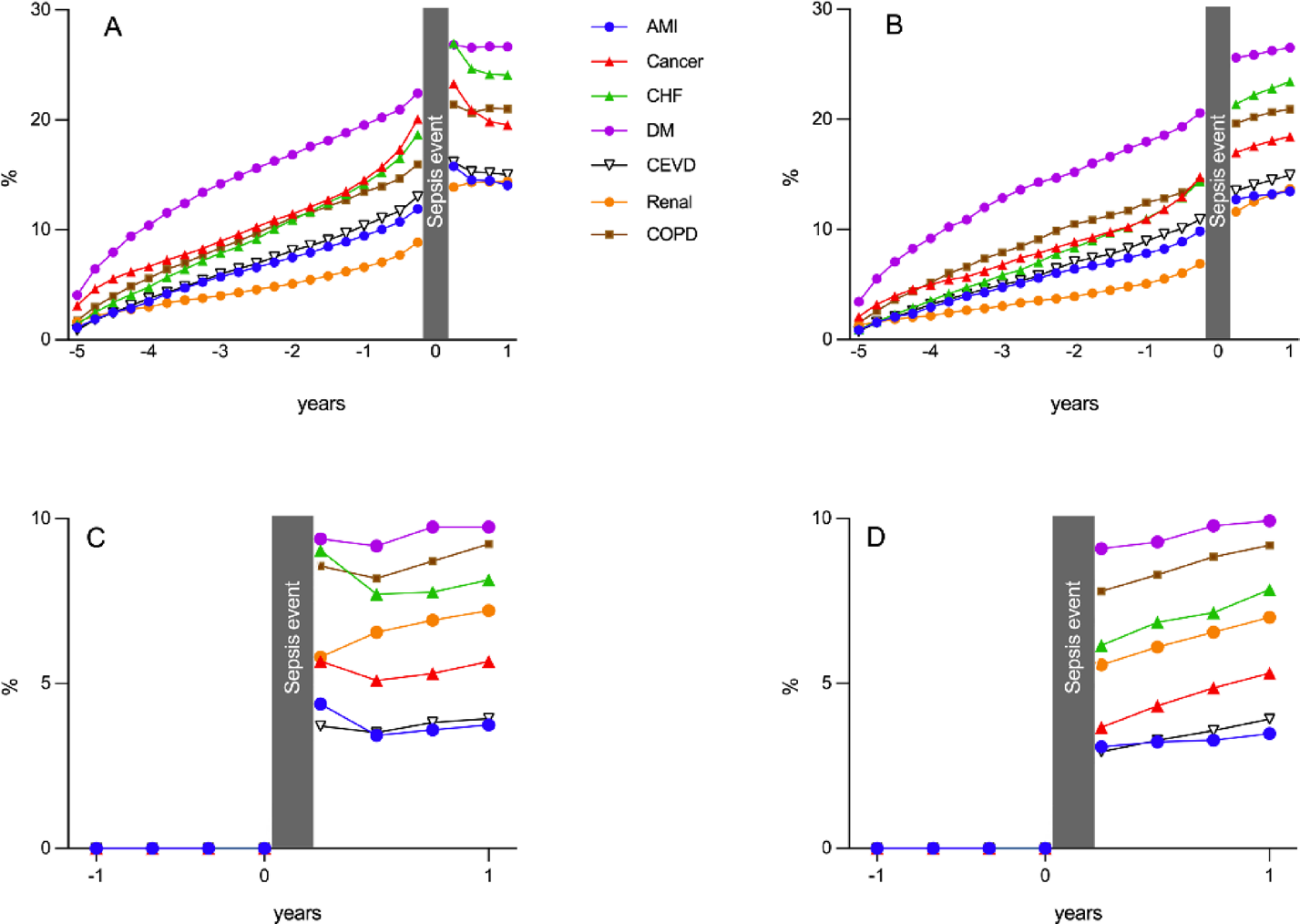
Trajectory of prevalence of the most common diagnoses included in Charlson’s Comorbidity Index over time. (**A**) Patients with sepsis. (**B**) Patients with sepsis surviving ≥ 1 year after admission. (**C**) Patients with sepsis with CCI zero before admission. (**D**) Patients with sepsis with CCI zero before admission surviving ≥ 1 year after admission. AMI (acute myocardial infarction), CHF (congestive heart failure), DM (diabetes mellitus), CEVD (cerebrovascular disease), Renal (renal disease) and COPD (chronic obstructive pulmonary disease).

 Diabetes was the most common diagnosis followed by congestive heart failure, chronic obstructive pulmonary disease (COPD) and cancer in the aftermath of ICU admission (see Fig. 2 A and B). For patients with CCI zero before ICU admission there was a notable increase in morbidity during the year after discharge (see Fig. 1 C and D). Diabetes, COPD, congestive heart failure, renal disease, and cancer were the most prevalent *de novo* diagnoses for these patients (see Fig. 2 C and D). The prevalence of the above diagnoses over time was lower among control individuals (see supplementary figure A-D). Cancer was the most common diagnosis among control individuals.

### Mortality

**Fig. 3:**
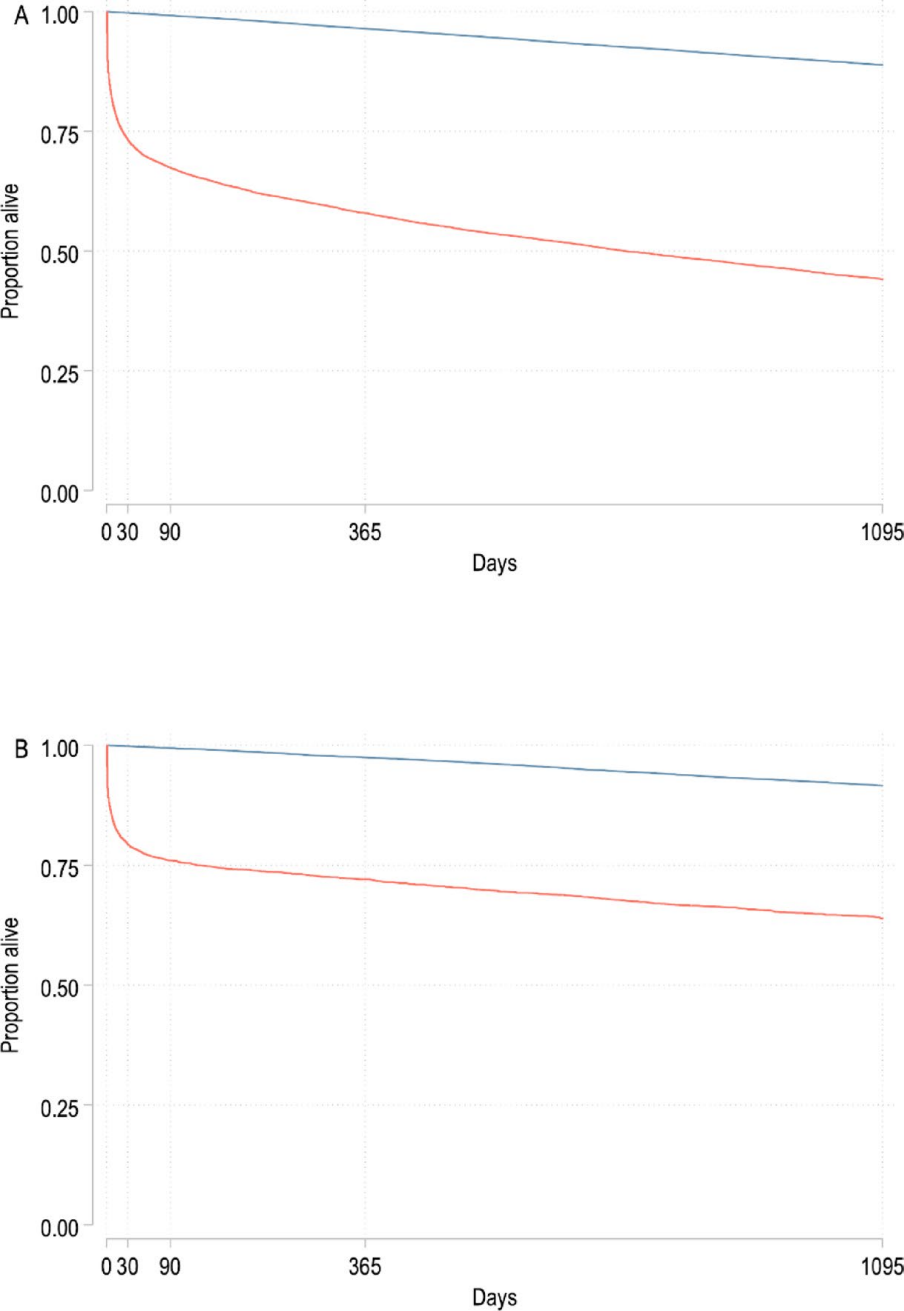
Kaplan-Meier survival plot. (**A**) Survival of patients with sepsis (red line) versus controls (dark blue line), P < 0.001 (by log rank test). (**B**) Survival of patients with sepsis with Charlson’s Comorbidity Index zero before admission and matched controls. P < 0.001 (by log rank test). Days from index date on x-axis.

 Patients admitted to the ICU for PCACS experienced considerable mortality. 32% had died by 90 days, 41% by one year, and 52% at three years (see Table 1; Fig. 3A). Additionally, for the subgroup of septic patients with CCI zero before admission, the mortality was significant. 24% had died by 90 days, 28% by 1 year, and 34% at 3 years (see Table 2; Fig. 3B).

**Fig. 4:**
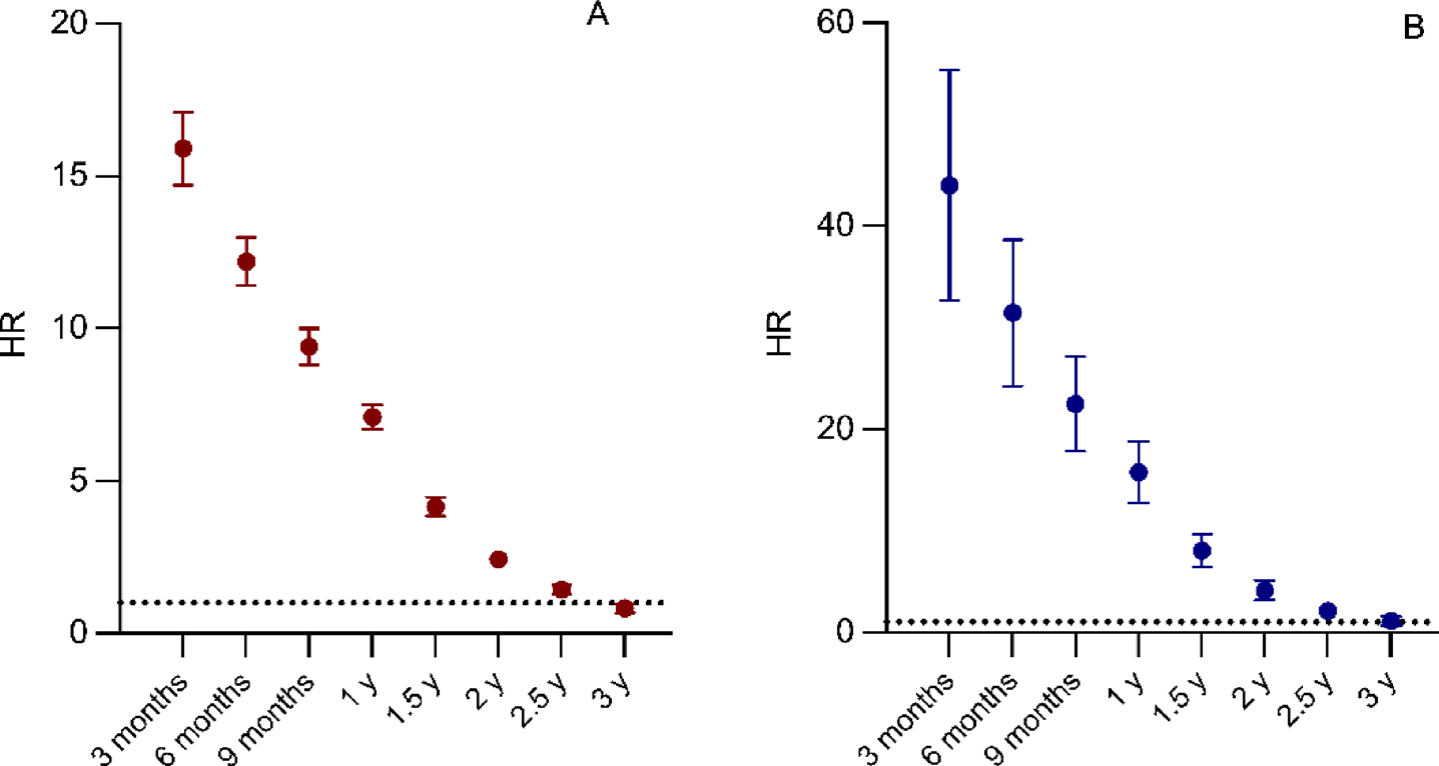
Hazard ratios for mortality over time for patients with sepsis versus controls. Y-axis hazard ratio (HR) with 95 per cent confidence intervals. X-axis time after index date. Analyses adjusted for Charlson’s comorbidity index (CCI), immunosuppression, income, education, psychiatric disease, and substance abuse at the time of the index date. (**A**) all patients with sepsis versus control individuals. (**B**) patients with sepsis and matched controls with CCI zero at the time of the index date.

Adjusted HRs for patients with sepsis compared with controls showed a sustained increased mortality up until three years after the index date. For the subset of patients with sepsis without prior somatic co-morbidity, even higher adjusted HRs were noted (see Fig. 4).

### Sepsis and risk of death among one-year survivors

There was an increase in morbidity among ICU survivors in the year following the septic event. In an exploratory analysis of one-year survivors, and adjusting for the burden of morbidity (CCI) at one year after the index date as well as other relevant confounders, exposure to PCACS remained associated with mortality in the period one to three years after the index date (unadjusted HR 4.4 (95% CI 4.1–4.8), adjusted HR 2.3 (95% CI 1.9–2.7)).

### Causes of death

**Fig. 5:**
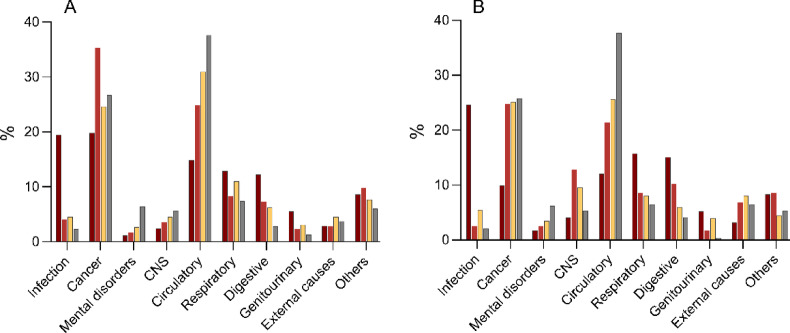
Proportions (%) of categories of causes of death for the patients with sepsis and controls. Time period 0–90 days depicted by dark red bars, 91 days to 1 year by red bars, 1–3 years by yellow bars for patients with sepsis. Proportions of causes of death for controls 0–3 years depicted by grey bars. A: all patients with sepsis versus control individuals. B: patients with sepsis with CCI zero before ICU admission versus their matched controls with CCI zero.

The pattern of causes of death changed during the follow-up period (see Fig. 5A). Among septic patients, the most common causes of early death (0–90 days) were infections (19%) and cancer (20%). Circulatory-, respiratory- and digestive causes accounted for 15%, 13% and 12%, respectively. Later causes of death (91 days-1 year and 1–3 years) were dominated by cancer and circulatory causes. These were also the most common causes of death among controls.

In the subgroup of patients with CCI zero before admission, infection was the most common cause of death in the early phase (25%), whereas cancer and circulatory causes also dominated the later phases (see Fig. 5B). The latter two were also the most common causes of death among the controls.

## Discussion

### Key findings

In this nationwide matched cohort study of patients admitted to the ICU for PCACS, we found a sustained excess mortality up until three-years after admission, even after adjusting for relevant confounders. A similar pattern was observed in patients without Charlson comorbidities before admission. The burden of comorbidity increased during the five years preceding ICU-admission and increased further during the following year. For patients with CCI zero before admission a notable increase in morbidity was seen during the year after ICU admission. Diabetes, congestive heart failure, and renal disease were among the most prevalent *de novo* diagnoses in these patients. When analysing the one-year survivors and adjusting for the burden of morbidity up to that time point as well as other clinically relevant confounders, exposure to PCACS remained associated with mortality for the period of one to three years after index date.

The pattern of causes of death changed during the follow-up period. As expected, infection was a common cause during the early phase, whereas cancer and circulatory causes dominated the later phases.

### Relationship with previous studies

The demographic characteristics of the sepsis cohort in our study align well with previous reports in terms of age, sex distribution and comorbidity load^22–24^. Notably, the overall burden of comorbidity was significant with more than half of the patients scoring two or more in CCI. The morbidity patterns observed in this study demonstrated a gradual increase over the five years preceding the septic event. This escalation was further intensified during the year preceding ICU admission, followed by an additional rise in the number of diagnoses during the post-discharge phase. The latter trend was notably conspicuous for patients with CCI zero on admission, where for example, the prevalence of diabetes, COPD, and congestive heart failure was almost ten per cent in the early post-discharge phase. These findings may suggest the presence of undiagnosed conditions before ICU admission, possibly contributing to the sepsis debut. Alternatively, these conditions may have emerged as a cause of sepsis per se or as the result of surveillance bias during hospital admission.

A noteworthy prevalence of congestive heart failure was observed in the post-discharge phase. The risk of cardiovascular events has been reported to be significantly increased in the aftermath of sepsis. In analogy with the findings of our study, a higher risk of subsequent cardiovascular events has been shown among sepsis survivors without cardiovascular disease before hospitalization^25^. In fact, a recent meta-analysis reported that sepsis may represent a long-term risk factor comparable to conventional cardiovascular disease risk factors such as hypertension, dyslipidaemia, and diabetes mellitus^26^. A proposed mechanism has been an accelerated vascular senescence induced by sepsis-related endothelial inflammation^27^. The prevalence of diabetes observed in the post-discharge phase among patients with a CCI of zero before admission prompts inquiry into causality: whether diabetes arises as a consequence of sepsis, or if the physiological strain of sepsis reveals previously latent diabetes. The latter aspect appears to contribute significantly to the explanation, as evidenced by a recent multi-centre study which reported a 13% prevalence of pre-diabetes or undiagnosed diabetes among patients admitted to the ICU^28^. The question of whether a severe infection may trigger a *de novo* onset of diabetes remains more elusive, although it has been suggested for SARS-CoV-2^29^.

The general interplay between sepsis and chronic morbidity is complex. A conceptual model posits a bidirectional relationship between chronic disease and sepsis. According to this model, chronic illness heightens the risk of sepsis development, while sepsis, in turn, may exacerbate the patient’s underlying chronic condition^30,31^.

A major finding in our study was the sustained association with excess mortality up until three years after ICU admission for sepsis patients. Interestingly, the adjusted hazards ratios for death were notably high for the subgroup of patients with CCI zero on admission. Several reports suggest that long-term mortality remains elevated over time for patients surviving sepsis, regardless of study settings^12,13,22,32–36^. The association with mortality raises the question of whether a causal relationship exists, though our observational design precludes such conclusions. Are these sustained mortality rates a consequence of sepsis itself, or are they a result of underlying morbidity or, in the case of patients with a CCI of zero, conditions emerging in the aftermath of sepsis? Overall, it is unclear whether late mortality after sepsis is predominantly driven by a continuing accumulation of morbidity or is also a result of sepsis per se. Previous attempts to explore this matter have been somewhat contradictory. Some reports, using morbidity data at admission, indicate that late mortality is a reflection of underlying comorbidity^37,38^, whereas others suggest it results from the sepsis itself^39^.

A large review of epidemiological studies exploring a potential link between exposure to sepsis and long-term mortality found the available literature of insufficient quality to sustain a consistent independent relationship^11^. A previous propensity score matched US study of septic patients reported that one in five deaths up to two years after admission could not be explained by health status before admission^36^. Another study of patients with sepsis under the age of 45 could not find an association between sepsis and late death beyond two years^34^. In an effort to investigate this relationship, we analysed the impact of sepsis on the risk of late mortality. In this exploratory analysis, which included patients and their matched controls surviving one year beyond the index date, we adjusted for pertinent confounders, encompassing the continuing burden of morbidity up to that one-year mark. In this analysis, exposure to sepsis was still associated with mortality during the period of one to three years after the index date, suggesting sustained effects of sepsis not entirely explained by the burden of morbidity after discharge. In this context, long-lasting post-sepsis immune dysfunction has been advocated as a contributing factor to late death^40^.

To our knowledge, the literature contains limited data on the causes of death after sepsis. As expected, infection was a dominant cause of death in the early phase although an equal proportion died of cancer. For the later causes of death, a majority were due to cancer and circulatory disease, consistent with a previous report on ICU-admitted patients with sepsis^41^. This pattern was also seen among control individuals. For patients with a pre-admission CCI score of zero, infection emerged as the predominant cause of death in the early phase. Cancer was identified as a contributing factor in nearly 10% of cases during this phase, and it constituted the most common cause of death (35%) during the period from 90 days to 1 year, suggesting the potential presence of undiagnosed malignancy at the time of admission as a possible precursor to sepsis.

### Implication of study findings

As the number of individuals surviving sepsis presumably continues to rise, the long-term effects of this exposure pose a significant health challenge^2^. Our study highlights the increasing importance in acknowledging the post-septic burden and the risk of excessive morbidity and mortality among sepsis survivors.

### Strengths and limitations

The large, nationwide multicentre cohort of ICU-admitted patients with sepsis gives our study high external validity and generalisability. The well-validated health registries in Sweden and the matched study design further strengthen the internal validity. The study was based on a high-resolution data set linked to national health registers including morbidity coding that is considered to have high validity^42^. Demographic characteristics are much in line with previous studies of ICU patients with sepsis. The proportion of missing data was low (< 2%) and found for education and income variables only. This is less than the 5%, which has been suggested as the maximum upper acceptance limit for large datasets^43^. All citizens with a Swedish identity number are included in the Cause of Death Register, regardless of whether the death occurs in Sweden or abroad. Thus, the loss to follow-up is considered to be low (< 2%) regarding time to death and cause of death.

Limitations include the register-based design. Only patients admitted to ICU were included in the study population, and no comparisons were made with hospitalized septic patients not admitted to ICU. We chose to only have patients admitted from the emergency department to minimise the heterogeneity of the sepsis population, this could also limit generalisability. Potential misclassification cannot be ruled out in registry-based studies. The dataset includes all care episodes from hospitals and outpatient care (not classified as primary care) and conditions significant enough to influence management during hospitalisation are likely to be captured. Milder comorbidities managed exclusively in primary care could be missed. Furthermore, due to limitations in the registry data, we were unable to reliably determine the site of infection and to differentiate between sepsis and septic shock. The Charlson Comorbidity Index was chosen as it is the most widely used comorbidity measure in sepsis research, facilitating comparison with previous studies. Although well-validated and adapted to ICD-10, some of its original weightings, particularly for conditions such as cancer and AIDS, may not fully reflect current prognostic relevance in ICU populations due to advances in treatment. Psychiatric illness and substance abuse were added as separate covariates, as these clinically relevant conditions are not captured by the CCI.

## Conclusions

Among patients admitted to the ICU for PCACS, there was a notable increase in morbidity during the period preceding admission, followed by a distinct rise in the post-discharge phase. Exposure to sepsis was associated with excessive mortality up until three years after ICU admission. For one-year survivors, sepsis remained associated with mortality, even after adjusting for the burden of morbidity up to that one-year mark, suggesting persistent long-term effects of sepsis not fully explained by diagnosed morbidity. Our study underscores the significance of recognising the post-septic burden and the risk of heightened morbidity and mortality among sepsis survivors.

## Supplementary Information

Below is the link to the electronic supplementary material.

Supplementary Material 1

Supplementary Material 2

Supplementary Material 3

## Data Availability

The datasets generated and analysed during the current study are available from the corresponding author upon reasonable request.
