# Supplementary figures and images for: The impact of community-acquired critical sepsis on long-term mortality and morbidity—a nationwide cohort study
Source: Sci Rep. 2026 May 20;16:15705. doi: 10.1038/s41598-026-53619-9 (PMC13187140; doi:10.1038/s41598-026-53619-9)

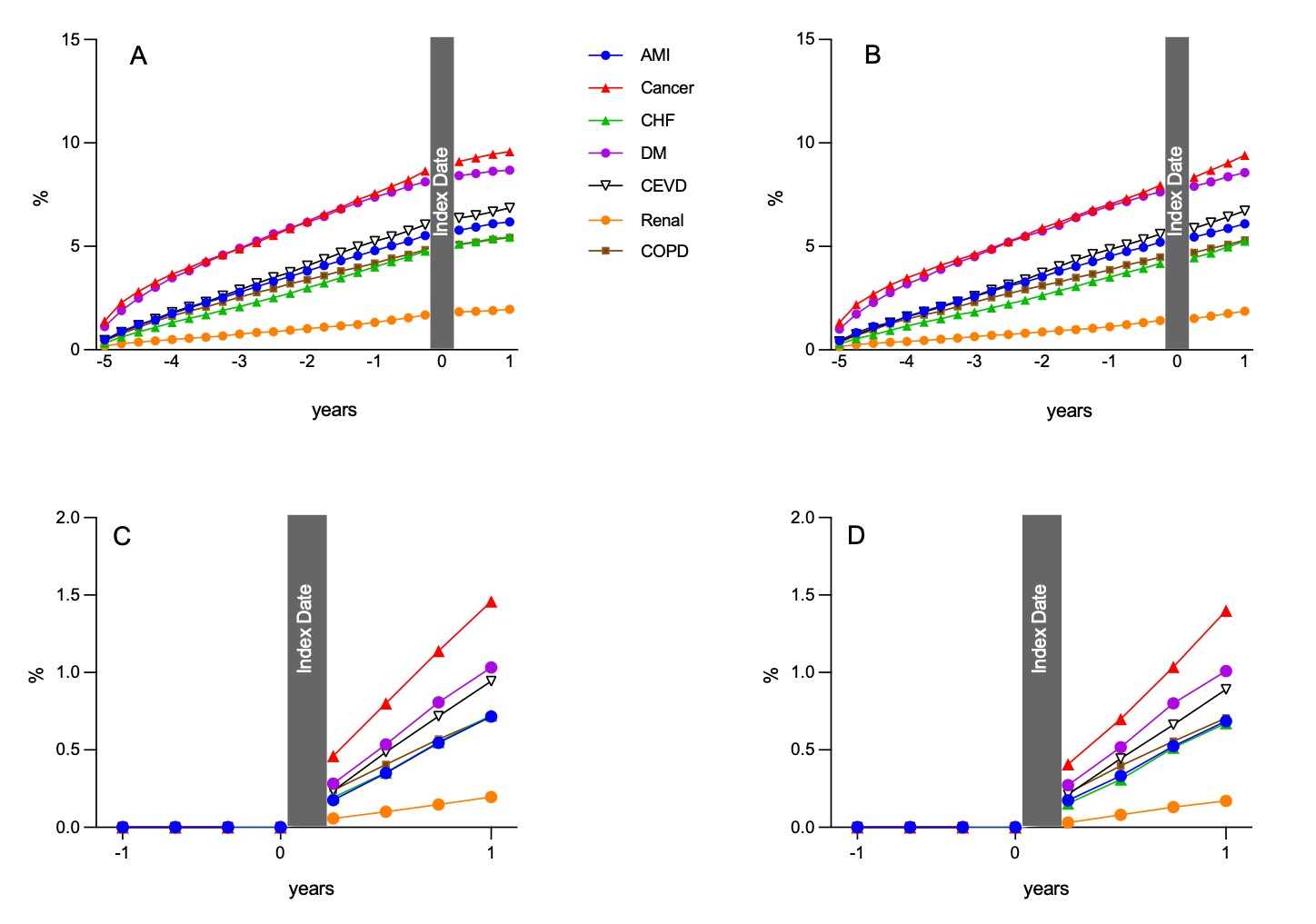

Supplement: Supplementary file 1 — Supplementary Material 1 [file 41598_2026_53619_MOESM1_ESM.jpg]
