# Supplementary material for: The impact of community-acquired critical sepsis on long-term mortality and morbidity—a nationwide cohort study
Source: Sci Rep. 2026 May 20;16:15705. doi: 10.1038/s41598-026-53619-9 (PMC13187140; doi:10.1038/s41598-026-53619-9)
Supplement: Supplementary file 2 — Supplementary Material 2 [file 41598_2026_53619_MOESM2_ESM.docx]

*Supplemental table*

|  | **Controls** | **Sepsis patients** |
| --- | --- | --- |
|  | n=50,180 | n=10,072 |
| Diabetes | 8% (4,076) | 22% (2,259) |
| Malignancy | 9% (4,336) | 20% (2,024) |
| Congestive heart failure | 5% (2,395) | 19% (1,881) |
| Chronic Obstructive Pulmonary disease | 5% (2,423) | 16% (1,607) |
| Cerebrovascular disease | 6% (3,033) | 13% (1,310) |
| Acute myocardial infarction | 6% (2,770) | 12% (1,199) |
| Renal disease | 2% (843) | 9% (893) |
| Peripheral vascular disease | 2% (1,208) | 9% (890) |
| Rheumatoid Disease | 2% (1,111) | 8% (796) |
| Liver disease | 1% (369) | 6% (653) |
| Hemiplegia or Paraplegia | 1% (364) | 4% (430) |
| Psychiatric disease | 5% (2,628) | 15% (1,525) |
| Substance abuse | 2% (812) | 9% (930) |

*Supplemental table depicting the distribution of comorbidities before ICU admission among control individuals and sepsis patients.*
