# Supplementary material for: The impact of community-acquired critical sepsis on long-term mortality and morbidity—a nationwide cohort study
Source: Sci Rep. 2026 May 20;16:15705. doi: 10.1038/s41598-026-53619-9 (PMC13187140; doi:10.1038/s41598-026-53619-9)
Supplement: Supplementary file 3 — Supplementary Material 3 [file 41598_2026_53619_MOESM3_ESM.docx]

*Supplementary figure. Trajectory of prevalence of the most common diagnoses included in Charlson’s Comorbidity Index over time for control individuals. A: All controls. B: Controls surviving ≥ 1 year after the index date. C: Controls with CCI zero before the index date. D: Controls with CCI zero before index date surviving ≥ 1 year after the index date. AMI (acute myocardial infarction), CHF (congestive heart failure), DM (diabetes mellitus), CEVD (cerebrovascular disease), Renal (renal disease) and COPD (chronic obstructive pulmonary disease).*
